# *qTGW12a*, a naturally varying QTL, regulates grain weight in rice

**DOI:** 10.1007/s00122-021-03857-4

**Published:** 2021-05-22

**Authors:** Zhixuan Du, Zhou Huang, Jianbin Li, Jianzhong Bao, Hang Tu, Chuihai Zeng, Zheng Wu, Haihui Fu, Jie Xu, Dahu Zhou, Changlan Zhu, Junru Fu, Haohua He

**Affiliations:** Key Laboratory of Crop Physiology, Ecology and Genetic Breeding, Ministry of Education, Research Center of Super Rice Engineering and Technology, Jiangxi Agricultural University, Nanchang, 330045 Jiangxi Province China

## Abstract

**Key message:**

**A stable QTL associated with rice grain type with a large effect value was found in multiple environments, and its candidate genes were verified by genetic transformation.**

**Abstract:**

Rice (*Oryza sativa* L*.*) grain size is critical to both yield and appearance quality. Therefore, the discovery and identification of rice grain size genes can provide pathways for the cultivation of high-yielding varieties. In the present work, 45,607 SNP markers were used to construct a high-density genetic map of rice recombinant inbred lines, and hence a total of 14 quantitative trait loci (QTLs) were detected based on the phenotypic data of grain weight, grain length and grain width under four different environments. *qTGW12a* and *qGL12* are newly detected QTLs related to grain weight, and are located between 22.43 Mb and 22.45 Mb on chromosome 12. Gene annotation shows that the QTL region contains the *LOC_Os12g36660* annotated gene, which encodes the multidrug and toxic compound extrusion (MATE) transporter. Mutations in exons and the splice site were responsible for the changes in grain type and weight. Gene knockout experiments were used to verify these results. Hence, these results provide a basis for the cloning of *qTGW12a*. This discovery provides new insights for studying the genetic mechanism of rice grain morphology, and reveals a promising gene to ultimately increase rice yield.

**Supplementary Information:**

The online version contains supplementary material available at 10.1007/s00122-021-03857-4.

## Introduction

As an important agronomic trait of rice, rice grain type is a principal index to measure the appearance quality of rice; moreover, it is also the key factor to affect rice yield. The main attributes reflecting grain type traits include grain length (GL), grain width (GW), grain thickness (GT) and grain aspect ratio (Ertao et al. 2008). Grain size is mostly measured by thousand-grain weight, which is a quantitative trait and is controlled by different genetic factors, such as the embryos, endosperm, and maternal plants. The grain type of rice is mainly determined by the glume, and the growth of the glume is regulated by cell numbers and size. The growth of glumes occurs mainly through cell division to increase the number of glume cells in the early stages of development. With the continuous growth of glumes, cell division is gradually be replaced by cell expansion (Li et al. 2018). Genetically, glumes are derived from diploid maternal plants, and their shape and size are controlled by the maternal genotype, while the endosperm, which accounts for the largest volume of seeds, develops from the triploid fertilized polar nucleus, and the embryo develops from a fertilized egg. Therefore, grain shape and size may be affected by both the maternal plant genotype and the zygotic genotype. In addition, cytoplasmic inheritance, such as the genetic material in chloroplasts or mitochondria, will also affect the development of grain.

From the perspective of heritability, the heritability of grain length and grain width is moderately high, and these traits are relatively stable under different environments, the heritability of grain thickness is low, with it being easily affected by the environment (Shi et al. 1999). With the development and application of molecular marker technology along with rice functional genomics and resequencing methods, using different genetic populations, more than 500 QTLs associated with rice grain size have been detected, and 93 rice grain size-related genes have been cloned (Wei et al. 2018). The number of clones distributed on 12 pairs of chromosomes differs, with the highest number on chromosome 3, and fewer on chromosomes 1, 9, and 12 (Huang et al. 2013). The QTLs that have been determined to control grain length and grain width mainly exhibit additive effects, but they also have a dominant effect. Grain thickness is mainly controlled by additive QTLs, which also influence maternal effects. The aspect ratio is a composite trait composed of grain length and grain width, and it is also mainly controlled by additive or dominant QTLs (Zuo and Li, 2014).

Rice grain development involves complex regulation, from the beginning of cell proliferation or elongation to the end of grain filling. This process entails plant hormones, the ubiquitin–proteasome pathway, the mitogen-activated protein kinase (MAPK) signaling pathway, the G protein signaling pathway, and epigenetic modification (Kesavan et al. 2013; Liu et al. 2015c, 2018; Zhang et al. 2016; Hu et al. 2018; Xu et al. 2018; Li et al. 2012). The genes involved in MAPK signaling cascade, G-protein signaling and the ubiquitin–proteasome pathway influence rice size by controlling cell proliferation, while pathways involving plant hormones and epigenetic modification simultaneously affect cell proliferation and expansion, and ultimately control grain size (Sun et al. 2018). An increasing number of studies have shown evidence for an interactive regulatory relationship between different grain size regulatory pathways (Li and Li, 2016).

*GW2* was the first gene controlling rice grain width to be cloned and it encodes a RING-type protein with E3 ubiquitin ligase activity. The *gw2* mutant is functionally disabled, unable to transfer ubiquitin to the target protein. The substrate could not be specifically recognized and degraded, and subsequently activating the division of the glume cells and increasing the width of the glume (Song et al. 2007). *WTG1* encodes an otubain-like protease, which is homologous to human OTUB1 and has deubiquitinase activity. Overexpression of *WTG1* causes a more slender grain through cell expansion mechanisms (Huang et al. 2017). In addition, *GW5*, as a nucleus localization gene, that acts by ubiquitin proteasome pathway (Shomura et al. 2008; Weng et al. 2008). *BG1* is an auxin-responsive gene and stimulates glume cells elongation and division, resulting in regulates the organ volume of plants (Linchuan et al. 2015). Hu (Hu et al. 2015) cloned the rice grain length gene *GS2*, encoding a growth regulator *OsGRF4*, which plays a key role in cell division during grain development. *Dwarf1* (D1) encodes a G protein α subunit Gα. Mutations in the *D1* gene result in a small round grain phenotype in rice grains, and the sensitivity to brassinolide (BR) is reduced, indicating that D1-mediated G protein and BR signal transduction pathways through an important mechanism ( Wang et al. 2006; Miura et al. 2009). A negative regulator of grain size is *small grain 1* (*SMG1*), which encodes the rice mitogen-activated protein kinase kinase OsMKK4 that promotes cell division (Penggen et al. 2014). It has been reported that *Dwarf and Small Grains1* (*DSG1*) encodes a mitogen-activated protein kinase (OsMAPK6) homologous to Arabidopsis AtMAPK6, which has phosphorylation activity. Down regulation of *DSG1* produces smaller grains and lower thousand-grain weight (Liu et al. 2015c). Promoter region hypomethylation of the *related to abscisic acid insensitive3*/*viviparous1 6* (*RAV6*) gene is responsible for increasing leaf inclination and smaller grains (Zhang et al. 2015). Furthermore, *GW6a* has histone acetyltransferase activity (OsglHAT1), and causes an increase in the number of glume cells and results in increasing grain weight and yield. (Song et al. 2015). In rice, grain size is controlled by multiple genes, and exhibits interaction effect. For example, Gao found that *GS3* and q*GL3* have an additive effect on the regulation of rice grain length (Gao et al. 2015). *GW2* and *GW5* positively regulate the expression of *GS3*. At the same time, *GW5* inhibits the transcription of *GW2*, thereby reducing its expression (Yan et al. 2011). *GS3* can mask the influence of *GW5* on grain length, and *GW5* can mask the influence of *GS3* on grain width (Yan et al. 2011).

Although many genes that control rice grain size have been cloned, and these genes also involve multiple regulatory pathways, there is still insufficient information concerning the complex mechanism of grain size regulatory pathways and the correlation between different regulatory pathways. In this study, a recombinant inbred line (RIL) population was constructed by crossing 9311 and Changhui 121. Through resequencing the population, a high-density genetic map was constructed, and QTL mapping was performed for grain traits based on data under four environments. This study lays a foundation for comprehensively describing the regulatory mechanism of rice grain development and establishing an ideal grain size regulatory network.

## Materials and methods

### Plant materials

The RIL population of F_14_ generation was developed from the cross of 9311 (an *indica* cultivar and founder parents, pedigree: Yangdao4 × Yan3021) and Changhui 121 (a fragrant rice restorer line, pedigree: Yuexiangzhan × Xiangxian402), using single seed descendant method. The plants were cultivated at the experimental field of Jiangxi Agricultural University, Nanchang province (28°68′ N, 115°86′ E; long-day conditions) in 2018 and 2019 (Nanchang 2018 and Nanchang 2019), and planted at Hainan province (18°31′ N, 109°71′ E; short-day conditions) in 2018 and 2019 (Hainan 2018 and Hainan 019).

### Grain size related traits measurement

Thirty fully filled seeds were randomly selected to measure the grain length and width with a Vernier caliper at the mature stage. More than 200 fully filled were weighed with an electronic micrometer balance, with the measurements used to calculate the thousand-weight of the seeds. The average of three measurements was calculated.

### Total DNA extraction and QTL analysis

The extraction of rice genomic DNA was performed according to the CTAB method (Paterson et al. 1993). The qualified genomic DNA samples were sequenced using the Hiseq Xten system with PE150 sequencing strategy. Sequences obtained were compared using BWA software. SNP and InDel variations were produced by the GATK3.3.0 process after comparison. The high-quality SNP loci of the population were classified, and LepMap3.0 was used to divide the linkage groups and the consequent construct a genetic map. QTL IciMapping software was used to analyse the QTLs related to the grain size traits with the inclusive composite interval mapping (ICIM) model, and the LOD threshold was 3.0 based on a 1000-permutation test.

### Gene knockout vector construction

Referring to the information published on the CRISPR direct website (http://www.crisr.dbcls.jp), target sequences with low off-target probability and high specificity at the candidate gene locus were identified. The methodology was followed to obtain the primer Oligo sequence. Knock-out mutants were generated using the CRISPR/Cas9 system (Beijing Viewsolid Biotech Co., Ltd.), and the specific operation was carried out according to the kit instructions. The recombinant vectors were introduced into rice calli via *Agrobacterium*-mediated transformation. The knockout was performed in Zhonghua 11, which is a *japonica* rice variety. The primers used are listed in Table S1.

## Results

### Phenotypic performance of grain size traits

**Fig. 1:**
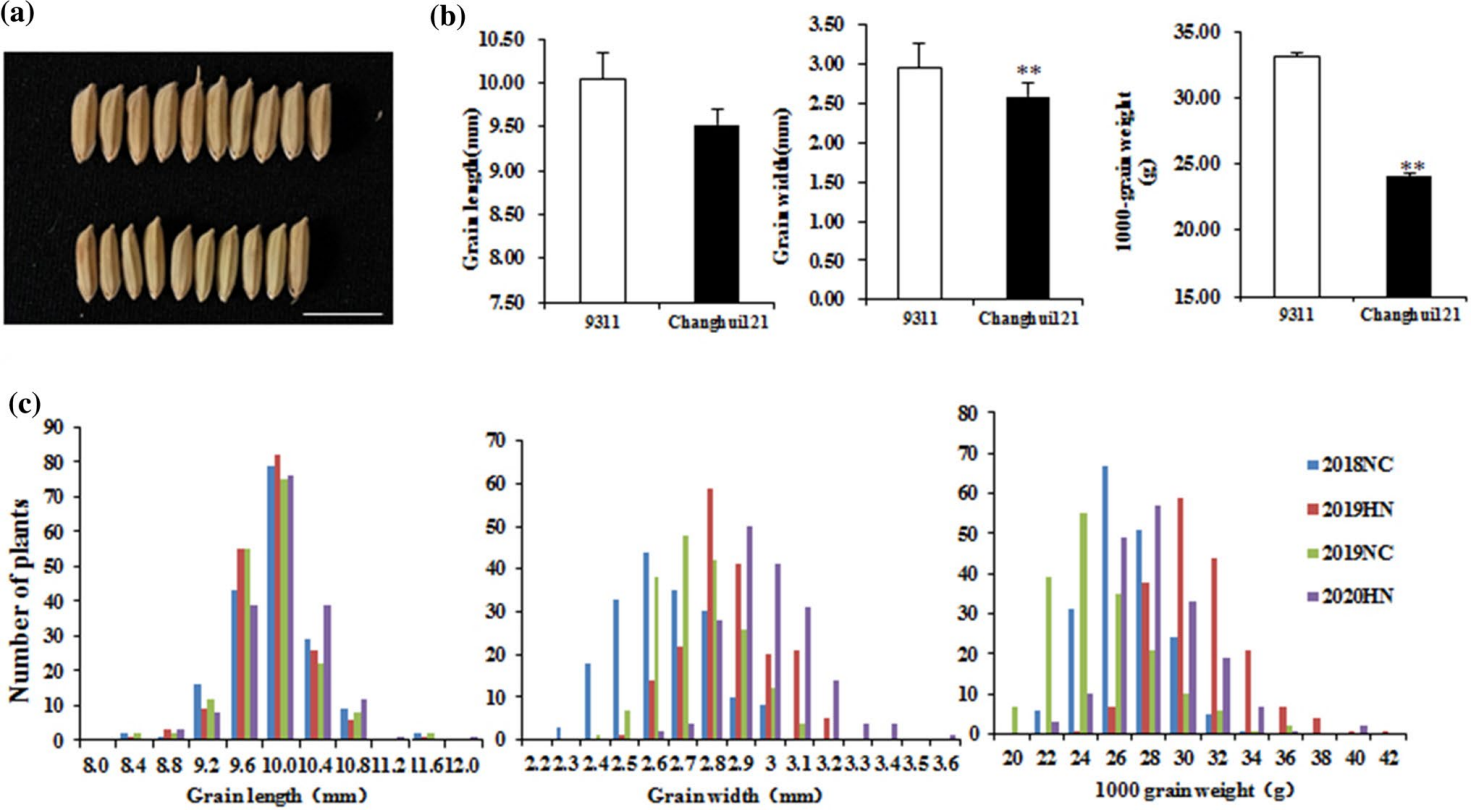
Grain size of two parents and frequency of the three grain traits in the RIL population. **a** Grain phenotypes of two rice parents 9311 and Changhui121, Bar, 1 cm. **b** Grain length, grain width and thousand-grain weight of 9311 and Changhui121. **c** The frequency of the three grain traits in the RIL population. Student’s t-test was used to generate *P* values; ** *P* < 0.01

The grain characteristics of the parents and the RILs population were studied under four types of environments (Nanchang in 2018, Hainan in 2019, Nanchang in 2019 and Nanchang in 2020) (Table 1). There were certain differences in the grain traits of 9311 and Changhui 121, including grain length, grain width and thousand-grain weight (Fig. 1a, b). The grain size data in the RIL population showed continuous and obvious separation under every environment, with a normal distribution, indicating that the grain size traits are regulated by multiple genes and were suitable for QTL analysis (Fig. 1c). In terms of grain length, there was no significant difference in the distribution of multipoint survey data for many years, and the frequency distribution trend was consistent. In terms of grain width, in 2019 and 2020, Hainan had an increase in grain width compared with that of Nanchang, with the normal curve and peak value shifted to the right. As it increased, the thousand-grain weight also changed consistently, demonstrating that the grain width and thousand-grain weight were significantly affected by the environment.

**Table 1:** Phenotypic values of grain size in recombinant inbred line populations

		Parents		RILs (*n* = 185)			
		9311	Changhui121	Mean	Range	Skewness	Kurtosis
GL (mm)	E1	10.45	9.70	9.77 ± 0.45	8.2–11.55	0.182	2.196
	E2	9.75	9.65	9.73 ± 0.39	8.1–11.4	−0.050	3.078
	E3	10.09	9.45	9.69 ± 0.44	8.3–11.58	0.544	3.563
	E4	9.85	9.31	9.80 ± 4.31	8.53–11.73	0.373	2.233
GW (mm)	E1	2.63	2.46	2.61 ± 0.16	2.25–3	0.133	−0.389
	E2	3.05	2.60^*^	2.85 ± 0.15	2.5–3.2	0.281	−0.405
	E3	2.94	2.53	2.70 ± 0.14	2.36–3.1	0.362	−0.153
	E4	3.21	2.76	2.93 ± 1.54	2.54–3.57	0.665	1.279
TGW (g)	E1	30.70	21.78**	25.83 ± 2.03	21.04–33.67	0.459	0.839
	E2	35.63	28.78**	29.82 ± 2.72	23.85–40.64	0.823	1.350
	E3	31.88	21.09**	24.07 ± 3.08	20.01–35.41	0.810	1.109
	E4	34.31	24.60**	27.36 ± 2.71	20.54–38.75	0.793	2.074

E1, Nanchang 2018; E2, Hainan 2019; E3, Nanchang 2019; E4, Hainan 2020. GL, grain length (mm); GW, grain width (mm); TGW, thousand-grain weight (g)

Student’s t-test was used to generate *P* values; * *P* < 0.05; ** *P* < 0.01

### Construction of genetic map

**Fig. 2:**
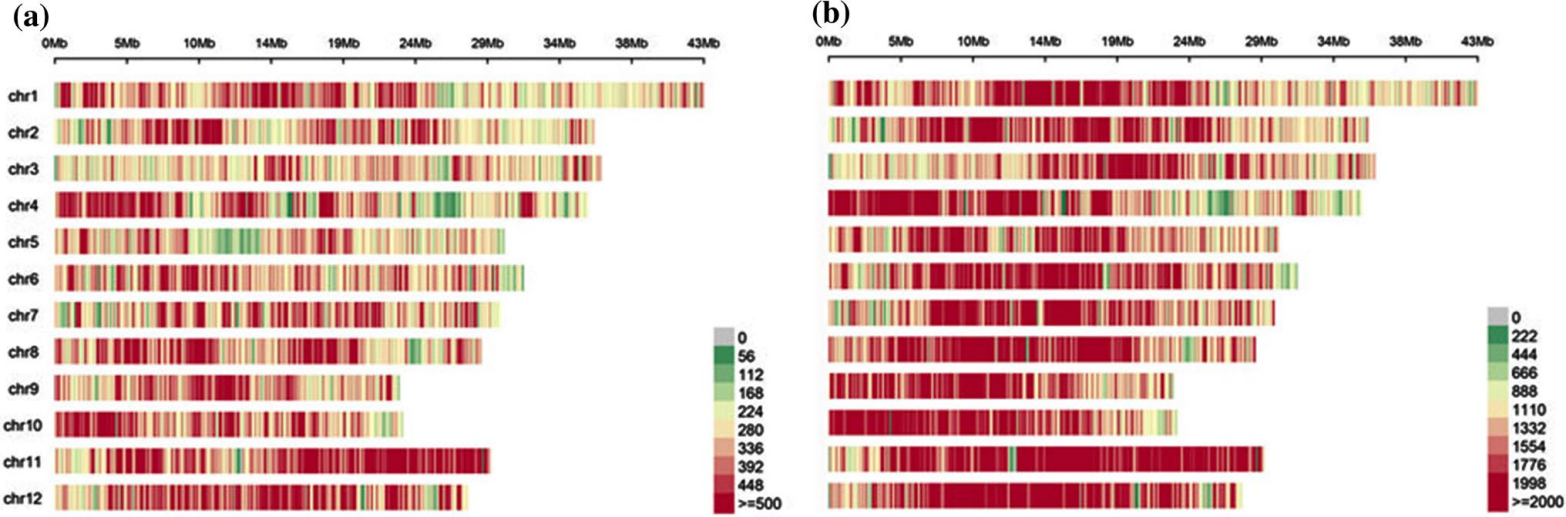
Distribution of SNPs and InDels on 12 chromosomes. **a** The number of InDels within 0.1 Mb window size. **b** The number of SNP within 0.1 Mb window size

**Fig. 3:**
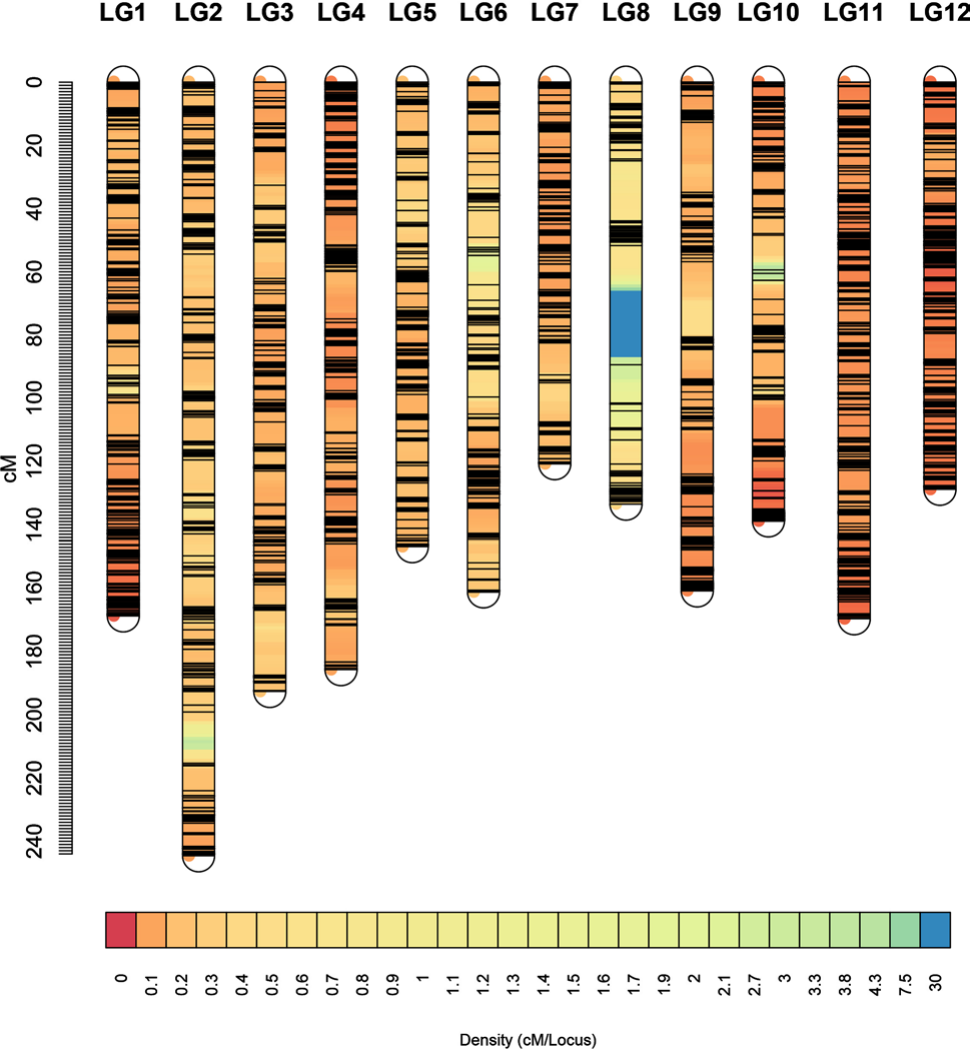
High density genetic map

Based on the re-sequencing of parents and RIL populations, a total of 6,658,585 initial SNPs and 1,371,642 initial InDel were produced. The statistical results of the distribution on each chromosome are as follows (Fig. 2). The genome was basically covered, and the overall distribution was even (Table S2), suggesting that the overall randomness of sequencing with a better quality. After filtering the initial population SNPs obtained, a high density genetic map was constructed using 45,607 SNP markers. A group of SNPs that were mapped to the same location constituted a bin, and the genetic map included 1,910 bin markers and distributed across 12 chromosomes. The total genetic distance was 1951.68 cM, and the average genetic distance between adjacent bin markers was 1.02 cM (Fig. 3).

In general, the bin markers were well distributed in the genome. The largest difference occurred in linkage group 02 (LG02), covering 244.56 cM, with 208 bin markers. The average distance between adjacent markers was 1.18 cM. The smallest LG was LG7, covering 120.72 cM, with 133 bin markers, and the average distance between adjacent marks was 0.91 cM (Table 2).

**Table 2:** Information of genetic linkage group

Linkage_map	SNP_marker	Bin_marker	Distance (cM)	Average_distance (cM)
LG1	5074	199	168.68	0.85
LG2	1678	208	244.56	1.18
LG3	288	148	192.63	1.30
LG4	5896	186	185.70	1.00
LG5	1323	147	146.77	1.00
LG6	1143	128	161.12	1.26
LG7	2206	133	120.72	0.91
LG8	203	87	133.52	1.53
LG9	3460	144	160.83	1.12
LG10	4975	131	138.77	1.06
LG11	7910	215	169.59	0.79
LG12	9351	184	128.79	0.70

LG, linkage group

### QTL mapping for grain size

Combining the genetic map and the grain size data of the RIL population investigated for four different environments, QTL mapping was performed. Using QTL IciMapping software with the inclusive composite interval mapping (ICIM) method, 14 QTLs related to grain size with large LOD were detected, and distributed on 6 chromosomes. Among them, there were 4 QTLs affecting grain length, which were distributed on chromosomes 1, 3 and 12, with a total contribution rate of 79.35%. *qGL12* had the largest contribution rate of 34%. Six QTLs distributed on 4 chromosomes were identified as affecting grain width, and the total contribution rate was 60.35%. There were 4 QTLs affecting the thousand-grain weight, and distributed on chromosomes 1, 2, 11 and 12 with a total contribution rate of 43.3% (Table 3).

**Table 3:** Stable QTLs related to GL, GW and TGW traits

	TraitName	Chr	LeftMarker	RightMarker	LOD	PVE (%)	Add	Candidate gene
GL	*qGL1-1*	1	1_6679372	1_7074285	21.7705	13.2919	0.1174	
	*qGL1-2*	1	1_7569739	1_7733586	26.9068	17.9592	−0.1138	
	*qGL3*	3	3_26719994	3_26723418	23.0879	14.0928	−0.2641	
	*qGL12*	12	12_22453339	12_22435836	46.9315	34.0029	0.191	
GW	*qGW2-1*	2	2_8279090	2_8154347	15.4991	12.7978	0.0363	*GW2*
	*qGW2-2*	2	2_26440999	2_26449189	4.3931	3.4574	0.015	
	*qGW3*	3	3_4028884	3_4241486	7.1212	5.6417	0.0229	*BG1*
	*qGW6*	6	6_24397171	6_24358768	6.5137	6.249	−0.0214	
	*qGW11*	11	11_26487110	11_23942040	24.2072	22.6255	1.0138	
	*qGW12*	12	12_25027080	12_24101810	9.773	9.5805	0.0262	
TGW	*qTGW1*	1	1_7109378	1_7483333	8.3845	16.1386	0.5547	
	*qTGW2*	2	2_8279090	2_8154347	5.2711	9.071	0.5094	*GW2*
	*qTGW11*	11	11_26487110	11_23942040	5.9683	11.2366	0.9875	
	*qTGW12a*	12	12_22453339	12_22435836	3.621	6.882	0.6947	

Among the QTLs associated with grain size, *qGW2-1* and *qTGW2* were mapped to the same interval of chromosome 2 and to coincide with the cloned grain width gene *GW2*. Therefore, the effect of *qGW2-1* and *qTGW2* may be caused by the major gene *GW2*. On the other hand, *qGL12* and *qTGW12a* are located in the interval of 22,435,836–22,453,339 on chromosome 12, indicating that there are two main QTLs affecting both grain size and thousand-grain weight in this region, suggesting that these QTLs had pluripotency. *qTGW12a* is not at the same locus as the QTLs related to grain size previously identified on chromosome 12 (*NAL3*, *OsPPKL3* and *OsSUT2*); hence, *qTGW12a* is the novel major QTL for regulating grain weight identified in this study.

### Candidate gene analysis of *qTGW12a*

**Fig. 4:**
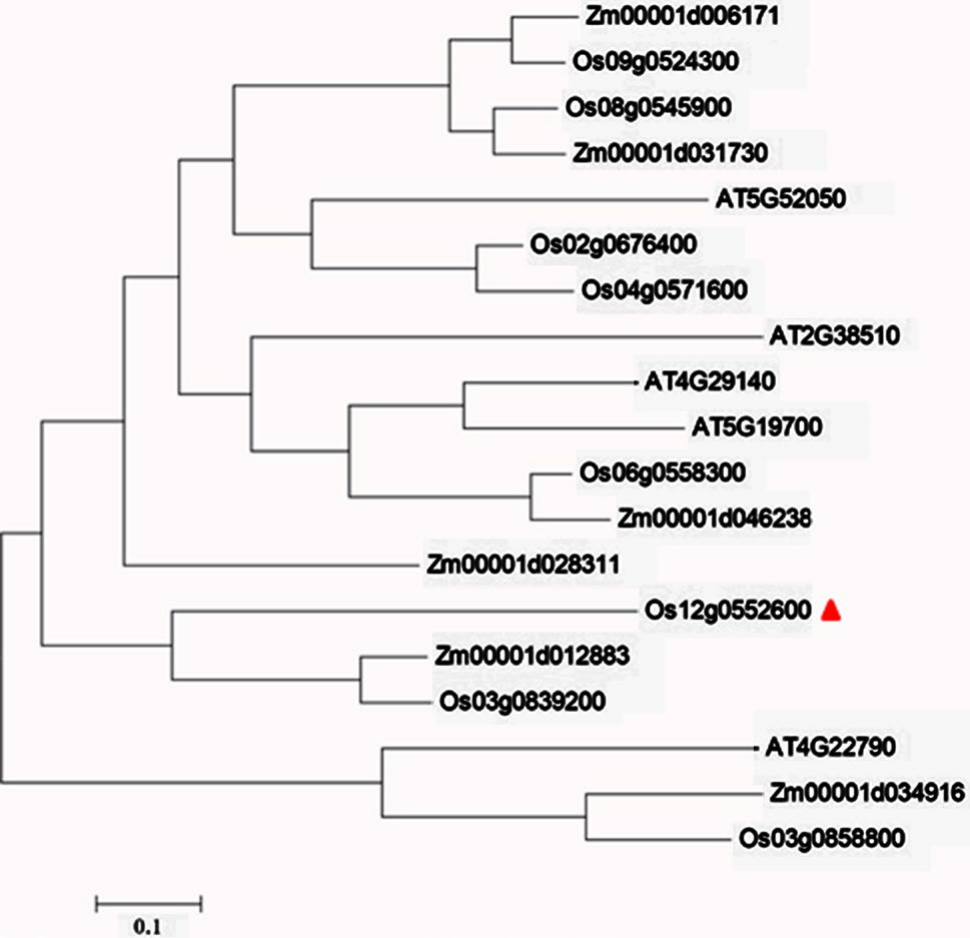
Phylogenetic tree of candidate gene and homologous proteins. The phylogenetic tree was constructed by MEGA 6.0 using the neighbor-joining method with 1000 replications

According to the MSU Rice Genome Annotation Project Database and Resource (http://rice.plantbiology.msu.edu/cgi-bin/gbrowse/rice/), there is a predicted gene in the region of 22,435,836–22,453,339 on chromosome 12 for *qTGW12a*: *LOC_Os12g36660*, which is 1498 bp in length, contains 2 exons and one intron, and encodes the multidrug and toxic compound extrusion (MATE) protein. Sequencing data showed that the *LOC_Os12g36660* (*Os12g0552600*) gene of Changhui 121 has 6 nonsynonymous mutations in the coding region, resulting in changes in encoded amino acids, as well as mutations (G → A) at the splicing site, causing an intron retention compared with 9311. The MATE proteins of rice, corn and Arabidopsis were analysed and found that that to be closely related to *Os03g0839200* in rice and *Zm00001d012883* (BIGE1) in maize (Fig. 4). The BIGE1 transporter is involved in regulating the size of seed organs. Therefore, *LOC_Os12g36660* was likely to be a candidate gene for *qTGW12a* and named *TGW12a.*

### Functional verification of *TGW12a*

**Fig. 5:**
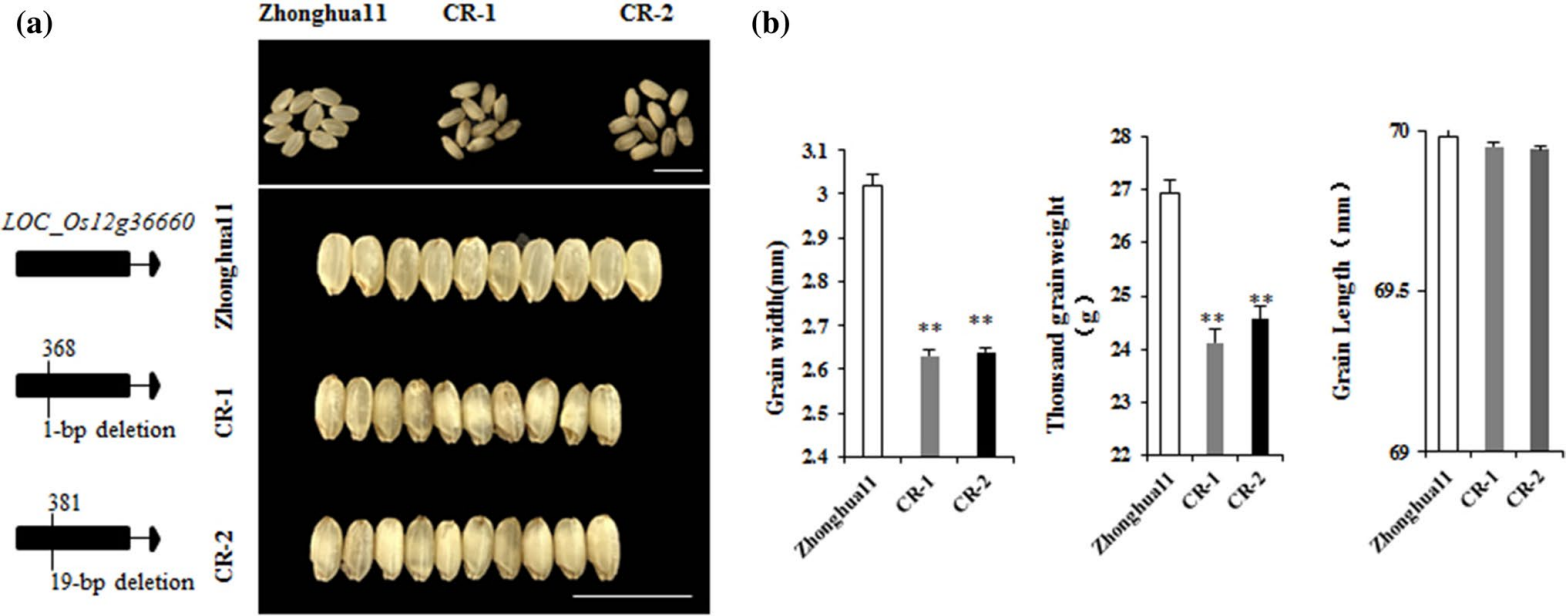
Gene editing and analysis of *TGW12a*. **a** Types of mutations and grain phenotypes. Bar, 1 cm. **b** Grain width, grain length and thousand grain weight of transgenic plants with mutated LOC_Os12g36660-CR. Student’s t-test was used to generate *P* values; ** *P* < 0.01

To determine whether *TGW12a* is the gene that causes changes in grain weight, we conducted genetic modification verification. The *LOC_Os12g36660* gene was knocked out using the CRISPR/Cas9 system, and Zhonghua11 was transformed by the *Agrobacterium*-mediated method to obtain transgenic positive plants. Two independent mutations were constructed in Zhonghua11 (*CR-1*and *CR-2*), including a 19-bp deletion and a 1-bp deletion in the target region (Fig. 5a). The phenotypic investigation showed that the grain size of the progeny of *LOC_Os12g36660*^*ZH11*^ knock-out plants was significantly narrower than that of the wild type, and the allele of *TGW12a* was responsible for the lower thousand-grain weight (Fig. 5b). It is further proven that the change in grain size was caused by editing *LOC_Os12g36660*. The mutation of *TGW12a* altered the grain size, but potentially due to the genetic compensation effect of the *TGW12a* mutations (resulting in altered expression of its homologs), their effect on the production of grain length was not as drastic as a knockout mutation would be expected to be. Consequently, knockout mutations of *TGW12a* eventually lead to a reduction in grain weight.

At the same time, the appearance quality of the transgenic rice lines was also affected, and obvious increased was observed in area of chalky endosperm. Hence, it is speculated that this gene may be involved in different regulatory pathways.

## Discussion

Yield has always been one of the main goals of rice breeding, but it is a complex trait that is affected by multiple factors. Grain size and panicle traits are two key traits determining grain yield in rice. The genetic basis of these traits is complex and controlled by multiple genes (Xia et al. 2018; Shi et al*.*, Shi et al. 2018; Ruan et al. 2020). With the rapid development of next-generation sequencing technology, through whole-genome resequencing, a bin-marker mapping based on chromosome fragments covering the genome has been perfomed and precise QTL positioning has been applied in many species (Liu et al. 2015a; Jiang et al. 2017; Luo et al. 2020). A large number of SNPs and InDel markers were detected by resequencing with the advantages of high genome coverage and high density, which can improve the accuracy of QTL mapping, and obtain relatively small chromosome reference intervals. For example, QTLs for plant height of foxtail millet using ultrahigh density genetic mapping were identified (He et al. 2021). Furthermore, QTLs for fiber quality traits under salt stress were obtained using a high-density genetic map (Guo et al. 2021).

In the present work, 14 QTLs related to grain size were identified. Comparing results of previous studies, it was determined that *qGW2-1* and *qTGW2* are the same site, and this interval contains the known major gene *GW2* (Song et al. 2007). Several studies have previously reported that *GW2* encodes a cyclic E3 ubiquitin ligase; the protein is localized to the cytoplasm and negatively regulates cell division in rice. Furthermore, the *qGW3* interval contains the cloned gene *Bg1*, *Bg1* is the original auxin response gene, and is involved in regulating the transport of auxin. It is a positive regulator of the auxin response and transport and affects cell division and cell elongation. Moreover, *Bg1* is a plant-specific regulatory factor that controls organ size (Liu et al. 2015b).

The candidate gene *TGW12a* encodes the MATE transporter. The MATE family members are cation secondary transporters, first obtained in the identification of multidrug efflux proteins from *Vibrio parahaemolyticus* and *E. coli* and mainly involved in the transmembrane transport of small molecule compounds (Debeaujon et al. 2001; Diener et al. 2001; Rogers and Guerinot, 2002; Marinova et al. 2007). MATE is ubiquitous in bacteria and eukaryotes. As an increasing number of members of the MATE family of proteins have been identified, the understanding of this family of proteins has gradually improved; however, the function of most MATE proteins is still unclear. In plants, the processes that MATE family proteins participate in mainly include: transport of secondary metabolites, such as anthocyanins; detoxification of toxic compounds or heavy metals; regulation of plant disease resistance; iron translocation and aluminium detoxification (Gomez et al. 2011; Sun et al. 2011; Fujii et al. 2012), *TT12* gene is involved in anthocyanin transport(Marinova et al. 2007), *ALF5* and *ADP1* genes are involved in plant lateral root development (Diener et al. 2001; Li et al. 2014), *FDR3* is involved in iron transport and so on (Rogers and Guerinot, 2002). The Arabidopsis *ZRZ* gene is involved in transmitting leaf-borne signals and determines the rate of organ activation (Burko et al. 2011).

*Bige1* (*Zm00001d012883*), a MATE transporter gene encoded in the maize (*Zea mays*), is involved in the regulation of maize organogenesis and organ size. Grain sizes developed homozygous mutant *Bige1* maize plants were also smaller than those of wild type plants. *Bige1* is located in the trans-Golgi apparatus, indicating that it may be involved in the secretion of signal molecules (Suzuki et al. 2015). The specific down regulation of the *OsMATE2* gene results in significantly smaller grain size along with early flowering and maturation in rice. Recently, Qin et al. cloned a gene, *DG1*, that regulates rice grain filling, encoding a MATE transporter. It has been illustrated that leaf-derived ABA controls rice seed development in a temperature-dependent manner and is regulated by *DG1* (Qin et al. 2021). Therefore, it is speculated that *TGW12a* also has a similar function to *BIGE1* and *OsMATE2* and it participates in the regulatory pathway of cell development.

The CRISPR/Cas9 targeted mutagenesis performs site-specific editing of the target gene and generates different types of allelic mutations. This method has been applied in transgenic verification experiments for multiple QTLs associated with rice grain size. For example, Wang knocked out the annotated gene adjacent to *GL7* and found that this gene can negatively regulate *GL7* (Wang et al. 2015). After the candidate genes *TGW3* and *GS9*, which control the grain length, were knocked out, the grains of the transgenic plants were changed (Ying et al. 2018; Zhao et al. 2018). In the present study, with the knockout verification test, the grain width and weight of the mutant were significantly decreased compared to those of the wild type. This result provides strong evidence that *LOC_Os12g36660* is a candidate gene for *qTGW12a*. Notably, the previously mapped QTL, *qTGW12a*, was involved in regulating the function of grain length, while the grain width of the transgenic lines also changed. The difference in expression specificity may reflect the sub-functionalization of the partially redundant MATE gene of the Zhonghua11 homologue gene. Furthermore, we hypothesized that the motif of *TGW12a* may be regulated by multiple transcription factors.

Comparison of genomic DNA of *TGW12a* from 9311 and Changhui 121 showed that there were 6 SNPs and 1 splice-site mutations in the coding region of the gene. The relationship between the mutations in this region and the grain phenotype has been not determined. All SNPs may be considered necessary for phenotypic variation, which requires further verification by genetic complementarity experiments.

## Conclusion

Our results indicated the potential of the *TGW12a* gene in regulating grain size, and further research is needed to elucidate the molecular mechanism of this gene in rice. Simultaneously, the newly identified QTLs regulating grain size may provide a potential new approach to exploring rice grain synthesis. These results provide a basis for cloning a QTL that has an important contribution to rice grain size variation.

## Supplementary Information

Below is the link to the electronic supplementary material.

Supplementary file1 (DOCX 14 kb)

## References

[CR1] Burko Y, Geva Y, Refael-Cohen A, Shleizer-Burko S, Shani E, Berger Y, Halon E, Chuck G, Moshelion M, Ori N (2011). From organelle to organ: ZRIZI MATE-Type transporter is an organelle transporter that enhances organ initiation. Plant Cell Physiol.

[CR2] Debeaujon I, Peeters AJ, Leon-Kloosterziel KM, Koornneef M (2001). The *TRANSPARENT TESTA12* gene of Arabidopsis encodes a multidrug secondary transporter-like protein required for flavonoid sequestration in vacuoles of the seed coat endothelium. Plant Cell.

[CR3] Diener AC, Gaxiola RA, Fink GR (2001). Arabidopsis *ALF5*, a multidrug efflux transporter gene family member, confers resistance to toxins. Plant Cell.

[CR4] Ertao W, Jianjun W, Xudong Z, Wei H, Linyou W, Qun L, Lixia Z, Wei H, Baorong L, Hongxuan L (2008). Control of rice grain-filling and yield by a gene with a potential signature of domestication. Nat Genet.

[CR5] Fujii M, Yokosho K, Yamaji N, Saisho D, Yamane M, Takahashi H, Sato K, Nakazono M, Ma JF (2012). Acquisition of aluminium tolerance by modification of a single gene in barley. Nat Commun.

[CR6] Gao X, Zhang X, Lan H, Huang J, Wang J, Zhang H (2015). The additive effects of *GS3* and *qGL3* on rice grain length regulation revealed by genetic and transcriptome comparisons. BMC Plant Biol.

[CR7] Gomez C, Conejero G, Torregrosa L, Cheynier V, Terrier N, Ageorges A (2011). In vivo grapevine anthocyanin transport involves vesicle-mediated trafficking and the contribution of anthoMATE transporters and GST. Plant J.

[CR8] Guo AH, Su Y, Huang Y, Wang YM, Nie HS, Zhao N, Hua JP (2021). QTL controlling fiber quality traits under salt stress in upland cotton (Gossypium hirsutum L.). Theor Appl Genet.

[CR9] He Q, Zhi H, Tang S, Xing L, Wang S, Wang H, Zhang A, Li Y, Gao M, Zhang H, Chen G, Dai S, Li J, Yang J, Liu H, Zhang W, Jia Y, Li S, Liu J, Qiao Z, Guo E, Jia G, Liu J, Diao X (2021). QTL mapping for foxtail millet plant height in multi-environment using an ultra-high density bin map. Theor Appl Genet.

[CR10] Hu J, Wang Y, Fang Y, Zeng L, Xu J, Yu H, Shi Z, Pan J, Zhang D, Kang S (2015). A rare allele of GS2 enhances grain size and grain yield in rice. Mol Plant.

[CR11] Hu Z, Lu S, Wang M, He H, Sun Le, Wang H, Liu X, Jiang L, Sun J, Xin X, Kong W, Chu C, Xue H, Yang J, Luo X, Liu J (2018). A novel QTL q TGW3 encodes the GSK3/SHAGGY-like kinase OsGSK5/OsSK41 that interacts with OsARF4 to negatively regulate grain size and weight in rice. Mol Plant.

[CR12] Huang K, Wang D, Duan P, Zhang B, Xu R, Li N, Li Y (2017). WIDE AND THICK GRAIN 1, which encodes an otubain-like protease with deubiquitination activity, influences grain size and shape in rice. Plant J.

[CR13] Huang R, Jiang L, Zheng J, Wang T, Wang H, Huang Y, Hong Z (2013). Genetic bases of rice grain shape: so many genes, so little known. Trends Plant Sci.

[CR14] Jiang N, Shi S, Shi H, Khanzada H, Wassan GM, Zhu C, Peng X, Yu Q, Chen X, He X, Fu J, Hu L, Xu J, Ouyang L, Sun X, Zhou D, He H, Bian J (2017). Mapping QTL for seed germinability under low temperature using a new high-density genetic map of rice. Frontiers Plant Sci.

[CR15] Kesavan M, Song JT, Seo HS (2013). Seed size: a priority trait in cereal crops. Physiol Plantarum.

[CR16] Li J, Chu H, Zhang Y, Mou T, Wu C, Zhang Q, Xu J (2012). The rice HGW gene encodes a ubiquitin-associated (UBA) domain protein that regulates heading date and grain weight. PLoS ONE.

[CR17] Li N, Li Y (2016). Signaling pathways of seed size control in plants. Curr Opin Plant Biol.

[CR18] Li N, Xu R, Duan P, Li Y (2018). Control of grain size in rice. Plant Reprod.

[CR19] Li R, Li J, Li S, Qin G, Novák O, Pěnčík A, Ljung K, Aoyama T, Liu J, Murphy A, Gu H, Tsuge T, Qu L (2014). ADP1 affects plant architecture by regulating local auxin biosynthesis. PLoS Genet.

[CR20] Linchuan L, Hongning T, Yunhua X, Ronghui C, Fan X, Bin H, Chengzhen L, Jinfang C, Jiayang L, Chengcai C (2015). Activation of big grain1 significantly improves grain size by regulating auxin transport in rice. Proc Natl Acad Sci USA.

[CR21] Liu H, Niu Y, Gonzalez-Portilla PJ, Zhou H, Wang L, Zuo T, Qin C, Tai S, Jansen C, Shen Y, Lin H, Lee M, Ware D, Zhang Z, Lübberstedt T, Pan G (2015). An ultra-high-density map as a community resource for discerning the genetic basis of quantitative traits in maize. BMC Genom.

[CR22] Liu L, Tong H, Xiao Y, Che R, Xu F, Hu B, Liang C, Chu J, Li J, Chu C (2015). Activation of big grain1 significantly improves grain size by regulating auxin transport in rice. Proc Nat Acad Sci USA.

[CR23] Liu Q, Han R, Wu K, Zhang J, Ye Y, Wang S, Chen J, Pan Y, Li Q, Xu X, Zhou J, Tao D, Wu Y, Fu X (2018). G-protein betagamma subunits determine grain size through interaction with MADS-domain transcription factors in rice. Nat Commun.

[CR24] Liu S, Hua L, Dong S, Chen H, Zhu X, Jiang J, Zhang F, Li Y, Fang X, Chen F (2015). OsMAPK6, a mitogen-activated protein kinase, influences rice grain size and biomass production. Plant J.

[CR25] Luo X, Deng H, Wang P, Zhang X, Li C, Li C, Tan J, Wu G, Wang Y, Cheng Q, He H, Bian J (2020). Genetic analysis of germinating ability under alkaline and neutral salt stress by a high-density bin genetic map in rice. Euphytica.

[CR26] Marinova K, Pourcel L, Weder B, Schwarz M, Barron D, Routaboul JM, Debeaujon I, Klein M (2007). The Arabidopsis MATE transporter TT12 acts as a vacuolar flavonoid/H+—antiporter active in proanthocyanidin-accumulating cells of the seed coat. Plant Cell.

[CR27] Miura K, Agetsuma M, Kitano H, Yoshimura A, Matsuoka M, Jacobsen SE, Ashikari M (2009). A metastable DWARF1 epigenetic mutant affecting plant stature in rice. Proc Nat Acad Sci USA.

[CR28] Paterson AH, Brubaker CL, Wendel JF (1993). A rapid method for extraction of cotton (Gossypium spp.) genomic DNA suitable for RFLP or PCR analysis. Plant Mol Biol Rep.

[CR29] Penggen D, Yuchun R, Dali Z, Yaolong Y, Ran X, Baolan Z, Guojun D, Qian Q, Yunhai L (2014). SMALL GRAIN 1, which encodes a mitogen-activated protein kinase kinase 4, influences grain size in rice. Plant J.

[CR30] Qin P, Zhang G, Hu B, Wu J, Chen W, Ren Z, Liu Y, Xie J, Yuan H, Tu B, Ma B, Wang Y, Ye L, Li L, Xiang C, Li S (2021). Leaf-derived ABA regulates rice seed development via a transporter-mediated and temperature-sensitive mechanism. Sci Adv.

[CR31] Rogers EE, Guerinot ML (2002). FRD3, a member of the multidrug and toxin efflux family, controls iron deficiency responses in Arabidopsis. Plant Cell.

[CR32] Ruan B, Shang L, Zhang B, Hu J, Wang Y, Lin H, Zhang A, Liu C, Peng Y, Zhu L, Ren D, Shen L, Dong G, Zhang G, Zeng D, Guo L, Qian Q, Gao Z (2020). Natural variation in the promoter of TGW2 determines grain width and weight in rice. New Phytol.

[CR33] Shi CH, He CX, Zhu J, Chen JG (1999). Analysis of genetic effects and genotype X environmental interaction effects on appearance quality traits of Indica rice. Chin J Rice Sci.

[CR34] Shi C, Ren Y, Liu L, Wang F, Zhang H, Tian P, Pan T, Wang Y, Jing R, Liu T, Wu F, Lin Q, Lei C, Zhang X, Zhu S, Guo X, Wang J, Zhao Z, Wang J, Zhai H, Cheng Z, Wan J (2018). Ubiquitin specific protease 15 has an important role in regulating grain width and size in rice. Plant Physiol.

[CR35] Shomura A, Izawa TK, Ebitani T, Kanegae H, Konishi S, Yano M (2008). Deletion in a gene associated with grain size increased yields during rice domestication. Nat Genet.

[CR36] Song XJ, Huang W, Shi M, Zhu MZ, Lin HX (2007). A QTL for rice grain width and weight encodes a previously unknown RING-type E3 ubiquitin ligase. Nat Genet.

[CR37] Song XJ, Kuroha T, Ayano M, Furuta T, Nagai K, Komeda N, Segami S, Miura K, Ogawa D, Kamura T, Suzuki T, Higashiyama T, Yamasaki M, Mori H, Inukai Y, Wu J, Kitano H, Sakakibara H, Jacobsen SE, Ashikari M (2015). Rare allele of a previously unidentified histone H4 acetyltransferase enhances grain weight, yield, and plant biomass in rice. Proc Nat Acad Sci USA.

[CR38] Sun S, Wang L, Mao H, Shao L, Li X, Xiao J, Ouyang Y, Zhang Q (2018). A G-protein pathway determines grain size in rice. Nat Commun.

[CR39] Sun X, Gilroy EM, Chini A, Nurmberg PL, Hein I, Lacomme C, Birch PR, Hussain A, Yun BW, Loake GJ (2011). ADS1 encodes a MATE-transporter that negatively regulates plant disease resistance. New Phytol.

[CR40] Suzuki M, Sato Y, Wu S, Kang BH, McCarty DR (2015). Conserved functions of the MATE transporter BIG EMBRYO1 in regulation of lateral organ size and initiation rate. Plant Cell.

[CR41] Wang L, Xu YY, Ma QB, Li D, Xu ZH, Chong K (2006). Heterotrimeric G protein α subunit is involved in rice brassinosteroid response. Cell Res.

[CR42] Wang Y, Xiong G, Hu J, Jiang L, Yu H, Xu J, Fang Y, Zeng L, Xu E, Xu J, Ye W, Meng X, Liu R, Chen H, Jing Y, Wang Y, Zhu X, Li J, Qian Q (2015). Copy number variation at the GL7 locus contributes to grain size diversity in rice. Nat Genet.

[CR43] Wei X, Zeng ZF, Yang WF, Al E (2018). Advances in studies on genetic regulation of rice grain shape. J Anhui Agric Sci.

[CR44] Weng J, Gu S, Wan X, Gao H, Guo T, Su N, Lei C, Zhang X, Cheng Z, Guo X, Wang J, Jiang L, Zhai H, Wan J (2008). Isolation and initial characterization of GW5, a major QTL associated with rice grain width and weight. Cell Res.

[CR45] Xia D, Zhou H, Liu R, Dan W, Li P, Wu B, Chen J, Wang L, Gao G, Zhang Q, He Y (2018). GL3.3, a Novel QTL encoding a GSK3/SHAGGY-like Kinase, epistatically interacts with GS3 to produce extra-long grains in rice. Mol Plant.

[CR46] Xu R, Yu H, Wang J, Duan P, Zhang B, Li J, Li Y, Xu J, Lyu J, Li N, Chai T, Li Y (2018). A mitogen-activated protein kinase phosphatase influences grain size and weight in rice. Plant J.

[CR47] Yan S, Zou G, Li S, Wang H, Liu H, Zhai G, Guo P, Song H, Yan C, Tao Y (2011). Seed size is determined by the combinations of the genes controlling different seed characteristics in rice. Theor Appl Genet.

[CR48] Ying JZ, Ma M, Bai C, Huang XH, Liu JL, Fan YY, Song XJ (2018). TGW3, a major QTL that negatively modulates grain length and weight in rice. Mol Plant.

[CR49] Zhang S, Wu T, Liu S, Liu X, Jiang L, Wan J (2016). Disruption of OsARF19 is critical for floral organ development and plant architecture in rice (Oryza sativa L.). Plant Mol Biol Rep.

[CR50] Zhang X, Sun J, Cao X, Song X (2015). Epigenetic mutation of RAV6 affects leaf angle and seed size in rice. Plant Physiol.

[CR51] Zhao DS, Li QF, Zhang CQ, Zhang C, Yang QQ, Pan LX, Ren XY, Lu J, Gu MH, Liu QQ (2018). GS9 acts as a transcriptional activator to regulate rice grain shape and appearance quality. Nat Commun.

[CR52] Zuo J, Li J (2014). Molecular genetic dissection of quantitative trait loci regulating rice grain size. Annu Rev Genet.

