# Supplementary material for: qTGW12a, a naturally varying QTL, regulates grain weight in rice
Source: Theor Appl Genet. 2021 May 22;134(9):2767–76. doi: 10.1007/s00122-021-03857-4 (PMC8354980; doi:10.1007/s00122-021-03857-4)
Supplement: Supplementary file 1 — Supplementary file1 (DOCX 14 kb) [file 122_2021_3857_MOESM1_ESM.docx]

**Supporting Information**

**Table S1 PCR primers used in this study**

| Primer | Primer sequence | Description |
| --- | --- | --- |
| 12g60-1-F | TCCACGAACACGCTCCAAAT | sequencing |
| 12g60-1-R | TCAGCGAGATCCACAGGAAC |  |
| 12g60-2-F | GTCTACCTCCGCAGCAAGG | sequencing |
| 12g60-2-R | GTGCTCCGACGAGGTAGAAG |  |
| 12g60-3-F | GTCACTCACAACAAGCGTGC | sequencing |
| 12g60-3-R | ATGCGTTAAGTTATTTTCGGAGGT |  |
| 12g60-F | CCAACGTCACCGGCTACTC | sequencing |
| 12g60-R | GAGATCCACAGGAACGCCAG |  |
| G36660-1F | CAGGAGCGCGCCGCCGGCGAGCT | sequencing |
| G36660-1R | AACAGCTCGCCGGCGGCGCGCTC |  |
| G36660-2F | CAGGGGGCTCCAGCCCGAGCGCG | sequencing |
| G36660-2R | AACCGCGCTCGGGCTGGAGCCCC |  |
| G36660-3F | CAGGGCGAAGCTGGTCATGCACG | sequencing |
| G36660-3R | AACCGTGCATGACCAGCTTCGCC |  |
| GP12850-9296-F | CCAACGTCACCGGCTACTC | CRISPR/Cas9 |
| GP12850-9296-R | GAGATCCACAGGAACGCCAG |  |

**Table S2 Statistical results for the 187 population SNPs and InDels**

| Chr | SNP_num | InDel_num |
| --- | --- | --- |
| chr1 | 698,014 | 152,284 |
| chr2 | 591,288 | 126,210 |
| chr3 | 537,139 | 111,787 |
| chr4 | 621,326 | 126,544 |
| chr5 | 466,236 | 91,092 |
| chr6 | 546,530 | 109,852 |
| chr7 | 511,202 | 106,368 |
| chr8 | 563,609 | 111,424 |
| chr9 | 426,730 | 86,699 |
| chr10 | 467,098 | 94,776 |
| chr11 | 651,594 | 136,025 |
| chr12 | 577,819 | 118,581 |
